# Structure-Function Analysis of Rny1 in tRNA Cleavage and Growth Inhibition

**DOI:** 10.1371/journal.pone.0041111

**Published:** 2012-07-19

**Authors:** Natalie Luhtala, Roy Parker

**Affiliations:** 1 Cancer Biology Graduate Interdisciplinary Program, University of Arizona, Tucson, Arizona, United States of America; 2 Department of Molecular and Cellular Biology and Howard Hughes Medical Institute, University of Arizona, Tucson, Arizona, United States of America; Institute of Molecular and Cell Biology, Singapore

## Abstract

T2 ribonucleases are conserved nucleases that affect a variety of processes in eukaryotic cells including the regulation of self-incompatibility by S-RNases in plants, modulation of host immune cell responses by viral and schistosome T2 enzymes, and neurological development and tumor progression in humans. These roles for RNaseT2’s can be due to catalytic or catalytic-independent functions of the molecule. Despite this broad importance, the features of RNaseT2 proteins that modulate catalytic and catalytic-independent functions are poorly understood. Herein, we analyze the features of Rny1 in *Saccharomyces cerevisiae* to determine the requirements for cleaving tRNA *in vivo* and for inhibiting cellular growth in a catalytic-independent manner. We demonstrate that catalytic-independent inhibition of growth is a combinatorial property of the protein and is affected by a fungal-specific C-terminal extension, the conserved catalytic core, and the presence of a signal peptide. Catalytic functions of Rny1 are independent of the C-terminal extension, are affected by many mutations in the catalytic core, and also require a signal peptide. Biochemical flotation assays reveal that in *rny1*Δ cells, some tRNA molecules associate with membranes suggesting that cleavage of tRNAs by Rny1 can involve either tRNA association with, or uptake into, membrane compartments.

## Introduction

T2 ribonucleases are conserved nucleases found in all branches of life. In eukaryotic cells, T2 ribonucleases affect a variety of processes including the regulation of self-incompatibility by S-RNases in plants [1]–[4], modulation of host immune cell responses by viral and schistosome T2 enzymes [5]–[9], and neurological development [10] and tumor progression in humans [11], [12]. In these contexts, T2 ribonucleases can have both catalytic and catalytic-independent functions (reviewed in [13]). For example, the effects of RNASET2 in humans on tumor progression appear to be independent of its catalytic activity [11], [12]. In contrast, the catalytic activity of the RNASET2 ortholog in *Saccharomyces cerevisiae*, Rny1, is required for cleavage of tRNA and rRNA molecules [14].

Analysis of Rny1 in yeast suggests it also has properties analogous to those seen for other RNASET2 orthologs. For example, during oxidative stress, Rny1 is required for the production of tRNA and rRNA fragments [14]. Similarly, expressing human RNASET2 rescues tRNA cleavage in yeast strains lacking Rny1 [14], and zebrafish neurons deficient for RNASET2 and plants deficient for RNS2 accumulate rRNA [15], [16]. In addition, Rny1 is a glycosylated protein that can localize to vacuoles [14]. Glycosylation and acidic nuclease activity are typical of T2 ribonucleases, but whether glycosylation is required for activity is unknown. Finally, Rny1 affects cellular growth and sensitivity to stress independently of its nuclease activity [14]. This non-catalytic lethality resembles the ability of RNASET2 to suppress ovarian tumor establishment in its catalytically inactive form [11], [12] and parallels the capacity of certain catalytically mutant pestiviral T2 ribonucleases to elicit host immune cell depletion [7], [8].

Since Rny1 has a signal sequence and has been reported to primarily be a secreted or vacuolar localized protein [14], [17], an unresolved issue is how Rny1 and its RNA substrates interact in the cell [14]. Since an Rny1-GFP fusion protein shows reduced vacuolar signal during stress, one possibility is that Rny1 enters the cytoplasm to engage RNAs for cleavage during stress [14]. Precedent for this model comes from experiments showing that a predominant mitochondrial nuclease, Nuc1, exits mitochondria during stress to modulate nuclear and possibly cytoplasmic RNA degradation [18], and in mammalian cells, lysosomal cathepsins can be released to the cytosol during some responses leading to cell death (reviewed in [19]). Alternatively, or in a second mechanism, cytoplasmic RNA might enter the vacuole, where the T2 enzyme has been shown to localize during stress. Recently, Haud *et al.* presented evidence for the accumulation of rRNA within lysosomes with loss of RNASET2 in zebrafish neurons [15]. Thus, an unresolved issue is how compartmentation of Rny1 affects its function and access to RNA substrates.

Cleavage of tRNA is not unique to yeast and is conserved in eukaryotes as a response to specific stresses, producing tRNA cleavage products mapping primarily to the anticodon loop [14], [20]–[25]. In mammalian cells, these fragments inhibit translation and localize to stress granules [24], [26], [27], which are cytoplasmic untranslating mRNPs that can aggregate during stress (reviewed in [28]). Coupled with the fact that rRNA fragments accumulate during stress conditions that induce tRNA cleavage [20], [23], these data suggest the possible regulation of translation complexes and associated translating RNAs in a stress-specific manner by ribonucleases such as Rny1, and loss-of-function of these enzymes might impinge on cellular survival during stresses. Interestingly, the human RNASET2 has been reported to localize to P-bodies [29] although the significance of this localization remains to be determined.

To begin to understand how Rny1 functions in both catalytic and catalytic-independent manners we have analyzed the regions of Rny1 for their functional importance. We demonstrate that catalytic-independent inhibition of growth is a combinatorial property of the protein and is affected by a fungal-specific C-terminal extension, the conserved catalytic core, and the presence of a signal peptide. Catalytic functions of Rny1 are independent of the C-terminal extension, are affected by many mutations in the catalytic core, and also require a signal peptide. Biochemical flotation assays reveal that in *rny1*Δ cells, some tRNA molecules associate with membranes suggesting that cleavage of tRNAs by Rny1 can involve either tRNA association with, or uptake into, membrane compartments.

## Results

### Domain Organization of Rny1

**Figure 1:**
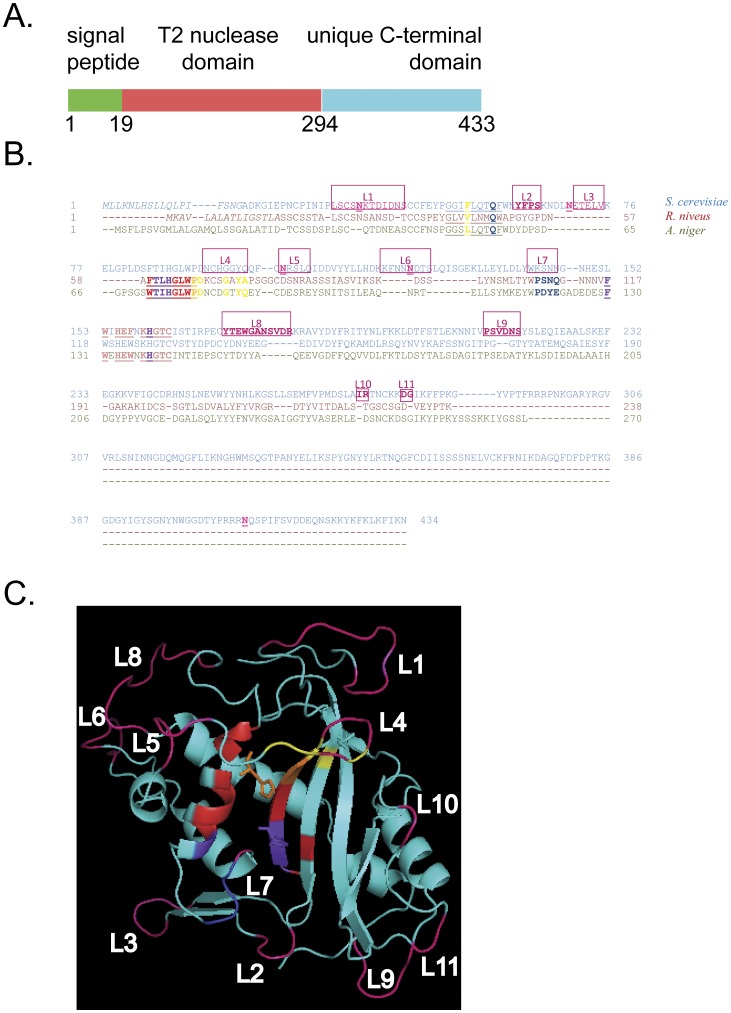
Sequence and structural analysis of Rny1. (A) Diagram indicating the positions within the amino acid sequence of *RNY1* regions analyzed by deletion. (B) COBALT alignment (http://www.ncbi.nlm.nih.gov/tools/cobalt/cobalt.cgi) of Rny1 of *S. cerevisiae* (top, in blue) to other ribonucleases of known structure (Rh, *R. niveus*, middle, in red; ACTIBIND, *A. niger*, bottom, in khaki) [31], [32]. T2 ribonuclease conserved amino acid sequences (CAS) are underlined and shown in red (I) and light red (II). Predicted nucleotide binding residues are shown in blue (B1 site) and yellow (B2 site) and are based on an alignment of Rh to ribonucleases whose structures are known in complex with nucleotides [33]. Residues that overlap involvement in B1 and CAS are shown in purple while those that overlap B2 and CAS are designated by orange (conserved sequence elements of T2 ribonucleases are reviewed in [13]). Putative N-linked glycosylation sites are depicted by underlined pink N residues which were identified by analysis with predictive glycosylation software (http://comp.chem.nottingham.ac.uk/cgi-bin/glyco/bin/getparams.cgi [37]). Loops targeted for mutation are boxed in pink and labelled L1–L10, corresponding to the structure in (C). Green and red circles above boxes indicate whether these loops were tested in active and/or inactive Rny1 backgrounds, respectively, with results of these analyses in Table 1. (C) Swiss Model predicted structure (Swiss Model (http://swissmodel.expasy.org/) was generated by 39% homology to ACTIBIND (de Leeuw, Roiz et al. 2007), and the image was illustrated in cyan using PyMol (www.pymol.org) with color coding and loop designations referring to those used in (A). Catalytic histidine residues are shown as protrusions within the T2 core in orange and purple. Loops L4 and L7 are predicted to participate in nucleotide binding based on our alignment to Rh which was previously aligned to ribonucleases with known regions of nucleotide binding (Rodriguez, 2008 #476).

Our general strategy was to make mutations in specific parts of Rny1 and examine their effects on catalytic and catalytic-independent functions. In this light, Rny1 possesses three domains (Figure 1A) defined in its initial characterization [17]. At its N-terminus it encodes a signal peptide (amino acids #1–18), presumably for insertion into the ER during translation. In the central region of the protein is a conserved RNaseT2 catalytic module (amino acids # 19–293). Within this region are critical amino acids known to be required for activity of RnaseT2 enzymes and for the nuclease activity of Rny1. For example, substitution of two catalytic histidine residues with phenylalanine (H87F and H160F) produces a catalytically inactive Rny1, referred to as rny1-ci [14]. Finally, the C-terminal region of Rny1 is a domain that is conserved in fungal species and is not seen in other eukaryotes [30].

To analyze the function of the conserved catalytic core, we desired to identify potential surface loops of amino acids that would not directly affect the folding of the protein, but might affect substrate interaction, or possibly protein-protein interactions. To do this, we took advantage of the high resolution structures of other T2 ribonucleases to predict possible loops for mutagenesis. Using Swiss Model (http://swissmodel.expasy.org/) and the known three-dimensional structure for the fungal T2 ribonuclease, ACTIBIND [31], we generated a predicted structure for Rny1 based on its 39% homology to this enzyme (Figure 1C). In addition, using COBALT (http://www.ncbi.nlm.nih.gov/tools/cobalt/cobalt.cgi), we aligned Rny1 to ACTIBIND and another fungal T2 ribonuclease, Rh, of known structure [32] (Figure 1B). This information revealed the position of eleven loops available for surface interactions, two of which (L4 and L7) could involve RNA binding based on previously published alignments of Rh to T2 ribonucleases with known nucleotide-bound structures [33]. In 10 of these loops, we generated mutants by replacing all loop residues with alanine (Table 1), generating mutations in catalytically active and/or inactive backgrounds. Contributions of these loops were examined in assays for growth-inhibition in wild-type and/or catalytically inactive backgrounds, and mutations in wild-type backgrounds were assayed for the ability to cleave tRNA.

**Table 1:** Effects of Rny1 mutants on growth and tRNA cleavage.

Name	Description	Growth	tRNA cleavage
Control	*GAL* vector	++++	–
WT	*GAL-RNY1*	+	++++
ci	*GAL-RNY1*, H87F, H160F	+	−
L2	aa 62–65, YFPS to AAAA	+	−
L3	aa 71–75, ETELV to AAAAA	+	−
L4	aa 93–99, NCHGGYQ to AAAAAAA	+	mobility shift
L5	aa 103–107, NRSLQ to AAAAA	+	NT
L6	aa 119–126, KFNNNDTS to AAAAAAAA	+	−
L7	aa 142–146, WKSNN to AAAAA	+	−
L8	aa 172–182, YTEWGANSVDR to AAAAAAAAAAA	+	NT
L9	aa 213–218, PSVDNS to AAAAAA	+	NT
L10	aa 273–274, IR to AA	+	++++
L11	aa 280–281, DG to AA	+	NT
ΔSP	aa 1–18, deleted	+++	+
ΔCTD	aa 294–433, deleted	++	++++
ΔT2	aa 19–293, deleted	++	−

Results of analysis of deletions to regions of Rny1 and alanine mutagenesis of loops 2–11 (L2–L11). Exact mutations are indicated for each construct. Growth was assayed by over-expression in the *hir2*Δ strain, using the rny1-ci background, on plates containing galactose. All strains were tested and grew equivalently on dextrose. Cleavage of tRNA was tested by harvesting *rny1*Δcells over-expressing the indicated constructs, resolving equivalent amounts of their total RNA by gel electrophoresis, and probing by Northern blot for tRNA Met(CAT). Additional details can be found within Materials and Methods. NT = not tested. aa = amino acids.

To analyze how either point mutations, or deletions to specific regions of Rny1 affect its function we used two assays. First, to assess the effect of a mutation on non-catalytic inhibition of growth, we examined how mutations impacted growth inhibition when Rny1 is over-expressed from the *GAL* promoter [14]. These experiments were done in a *hir2*Δ background, which we had seen can increase the growth inhibitory effects of Rny1 over-expression (data not shown). Second, to determine the effect of a mutation on nucleolytic function, we examined how a given Rny1 variant could restore tRNA fragment production to an *rny1*Δ strain.

### Analysis of Domains

**Figure 2:**
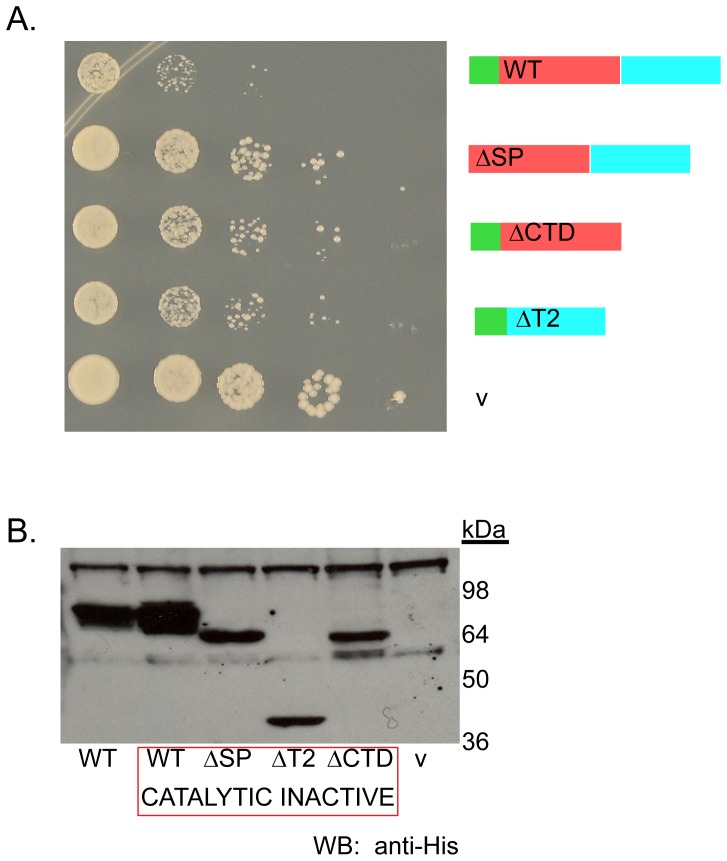
Multiple regions of Rny1 affect growth. (A) Frog ponds (performed as described in Materials and Methods section) on synthetic selective media plates containing galactose to induce Rny1’s over-expression in catalytic mutant background in a *hir2*Δ strain as a *GAL-RNY1* plasmid either full-length (WT), deleted for either the signal peptide sequence (ΔSP), the T2 conserved region (ΔT2) or the unique C-terminal region (ΔCTD) or a vector control (v). (B) Western blot (performed as indicated in Materials and Methods) of strains expressing constructs as shown in (A) except that the first lane shows a non-catalytic, full-length mutant GAL-RNY1’s expression in the same strain (WT). Migration of molecular weight standards is indicated.

We examined if the signal peptide, the catalytic core or the C-terminal extension was necessary or sufficient to inhibit cell growth when over-expressed. Moreover, to avoid any complications due to Rny1 nuclease activity, deletions of these regions were made in the context of the rny1-ci mutation. This led to the following key observations. First, we observed that deletion of the signal sequence reduced toxicity (Figure 2A). Second, we observed that deletion of either the central conserved core, or the C- terminal extension reduced, but did not abolish, toxicity (Figure 2A). These effects are not due to loss of protein expression since these variants were all expressed (Figure 2B). We interpret these observations to argue that nuclease-independent toxicity is a combinatorial property of both the central conserved core and the C-terminal extensions and requires the protein to contain a signal sequence.

**Figure 3:**
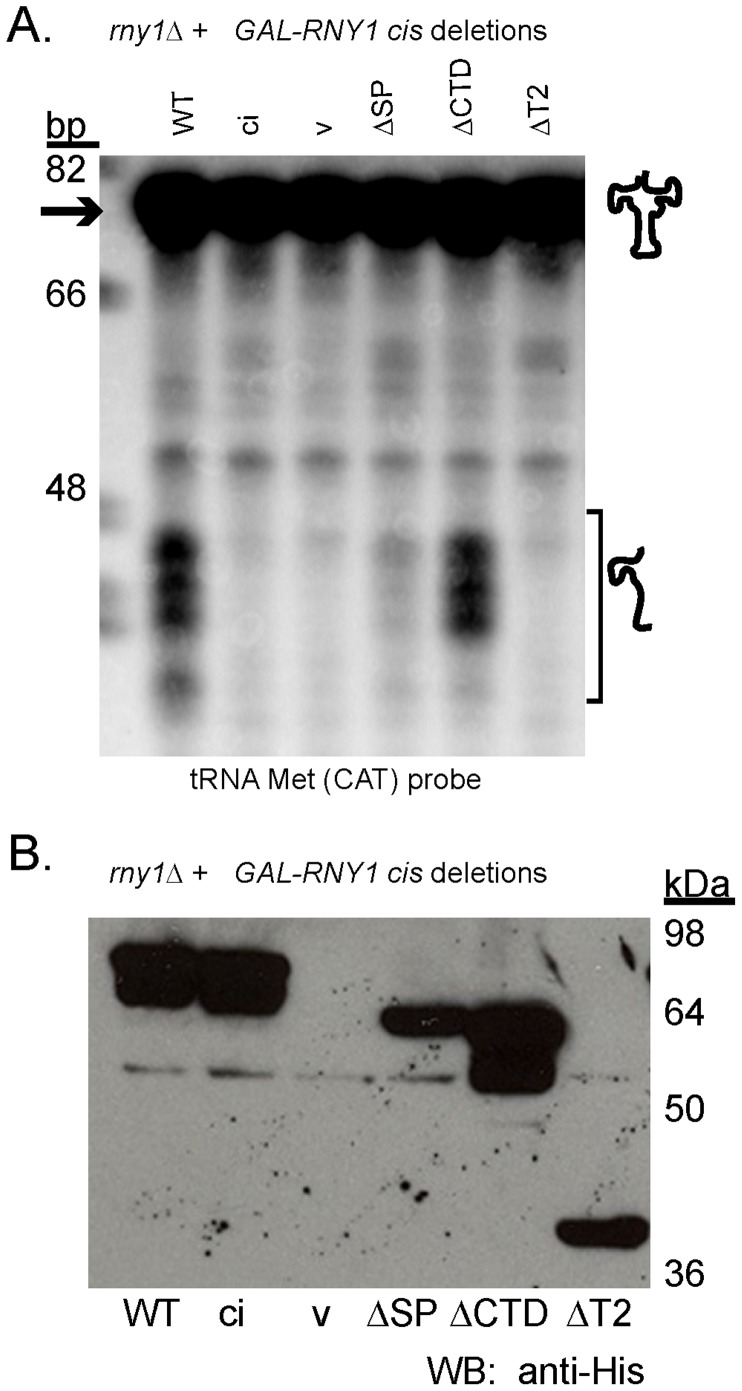
The signal peptide and T2 region affect tRNA cleavage. (A) Northern blot performed, blotting for tRNA Met(CAT), as detailed in Materials and Methods. Strains deleted for *RNY1* expressing *GAL-RNY1* mutant constructs (abbreviations defined in Figure 2) expressed in the catalytically active background. Migration of oligonucleotide standards is shown in base pairs (bp). (B) Western blot (performed as indicated in Materials and Methods) of strains expressing constructs as shown in (A). Migration of molecular weight standards is indicated.

We also examined the effects of these deletions on tRNA cleavage when Rny1 is over-expressed [14]. We observed that both the signal peptide and the central RNaseT2 domain were required for efficient tRNA fragment production, and their deletions resemble the phenotype of the rny1-ci allele (Figure 3A). In contrast, the C-terminal extension is not required (Figure 3A, ΔCTD lane). The ability to express proteins from the mutant constructs containing catalytic sequences was not lost (Figure 3B). We conclude that in addition to the catalytic core domain, a signal sequence is required for cleavage of RNA substrates by Rny1.

**Figure 4:**
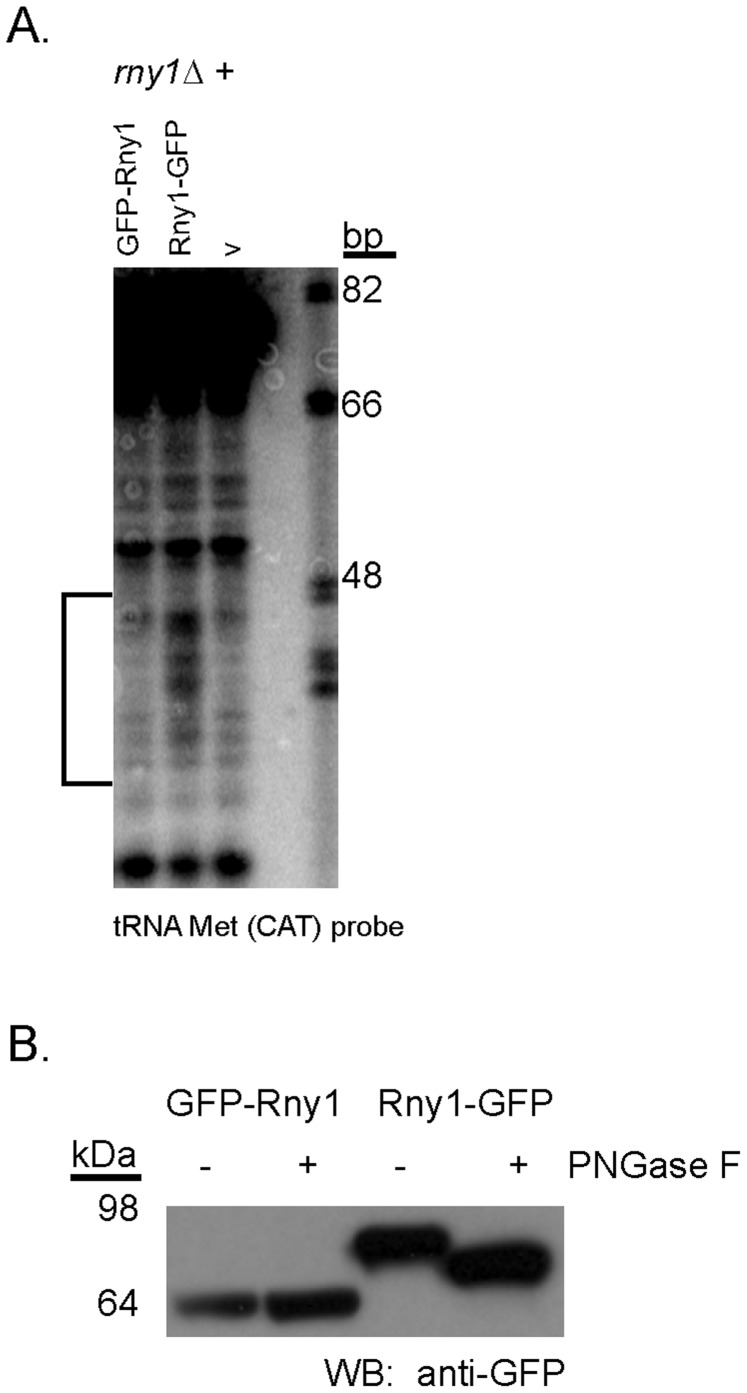
A glycosylation mutant lacks RNA cleavage activity. (A) Northern blot probing for tRNA Met(CAT). Strains deleted for *RNY1* expressing Rny1-GFP, GFP-Rny1, or vector. Migration of oligonucleotide standards is shown in base pairs (bp). (B) PNGase F or control digests of total lysates of wild-type strains expressing Rny1-GFP or GFP-Rny1. Samples were resolved by SDS-PAGE and probed by Western blot for GFP. Migration of molecular weight standards is indicated.

One possible interpretation of our results is that glycosylation might be important for Rny1’s functions. We analyzed Rny1-GFP fusion proteins where the GFP is either fused to the C-terminus of the protein or was inserted immediately after the signal peptide [14]. We observed that fusion of GFP to the C-terminal end of the protein (Rny1-GFP) still allowed inhibition of cell growth when over-expressed (data not shown), was able to restore tRNA fragment production in a *rny1*Δ strain (Figure 4A), and was glycosylated as judged by a reduction in molecular weight when treated with the endoglycosidase, PNGaseF (Figure 4B). In contrast, the fusion with GFP inserted just after the signal peptide failed to inhibit growth when over-expressed (data not shown), failed to restore tRNA fragment production to an *rny1*Δ strain (Figure 4A), and was not glycosylated (Figure 4B). These observations are consistent with the requirement for a signal peptide for function and support a model whereby Rny1 activity requires insertion of the nascent peptide into the ER and possibly glycosylation.

### Analysis of Mutations in the Catalytic Core Domain

**Figure 5:**
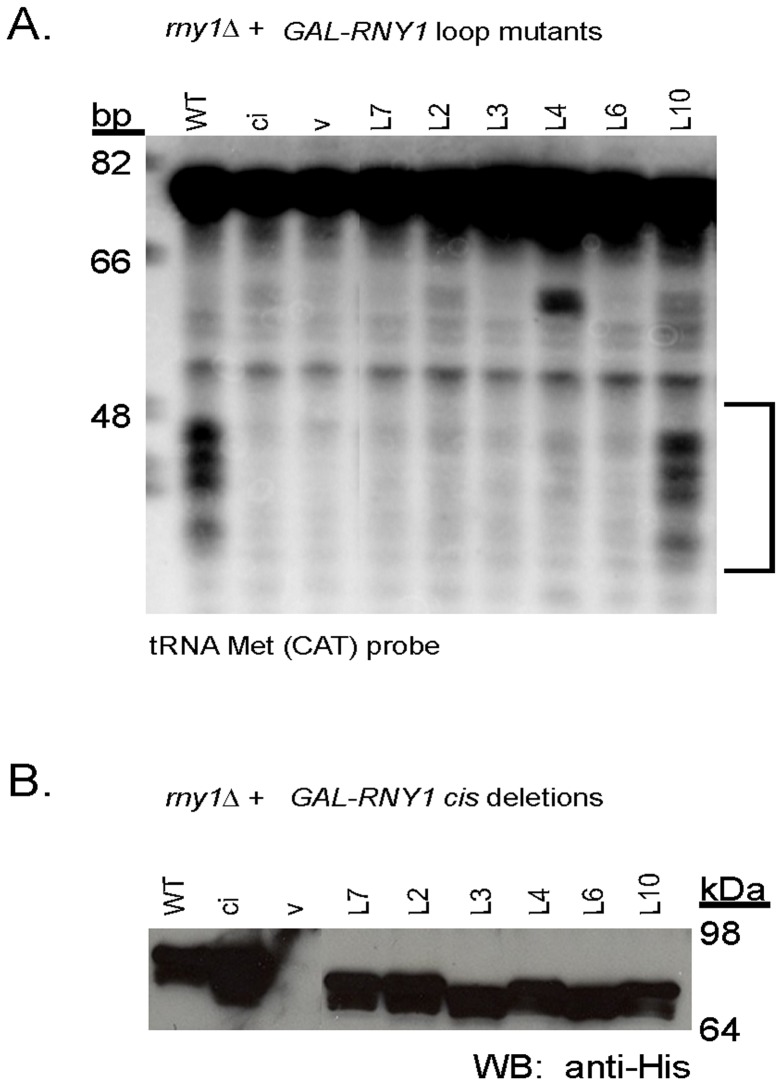
Multiple loops regulate RNA cleavage. (A) Cleavage of tRNA Met(CAT) by over-expressed full-length (WT), full-length catalytically inactive (ci), vector control (v), or RNY1 containing catalytic histidines but mutated in *cis* at indicated loops (L#). Strains deleted for *RNY1* were grown as described to induce plasmid over-expression of *GAL-RNY1* in the plasmid mutant or control indicated, and equivalent amounts of RNA were resolved and transferred to gels for Northern blots using oRP1401, all performed as indicated in the Materials and Methods. All samples are from the same image of the same blot, and the image was cropped after scaling the image to show only relevant samples (for A and B). Bracket reveals where expected bands accumulate with over-expression of the full-length (WT) *RNY1*. Migration of oligonucleotide standards is shown in base pairs (bp). (B) Western blot (performed as indicated in Materials and Methods) of strains expressing constructs as shown in (A). Migration of molecular weight standards is indicated.

We also analyzed how mutations in the loop regions of the core domain of Rny1 affected its function. We observed that none of the mutations in the loop regions reduced the ability of Rny1 to inhibit growth when over-expressed either in the active Rny1 or rny1-ci background (Table 1). This is consistent with the observations above (Figure 2) that growth inhibition is a combinatorial property of the whole protein. We also observed that mutations within loop 2, 3, 6, or 7 inhibited tRNA cleavage, while loop 10 was dispensable for this activity (Figure 5A) even though mutant proteins were all expressed at similar levels (Figure 5B).

A surprising observation was that mutations in loop 4, which are near a predicted RNA binding site alter the predominant cleavage product (Figure 5A). This is surprising since RNAseT2 enzymes are thought to be generally non-specific in their cleavage sites and to cleave tRNA predominantly in the anticodon loop since this is the most exposed part of the tRNA. One possibility is that the mutations in the L4 loop alter the positioning of the tRNA in the active site to preferentially lead to cleave at other sites in the tRNA.

Taken together, we suggest that specific loop regions in the catalytic core are required for Rny1’s catalytic activity and can play a role in determining the specific site of RNA cleavage.

### Rny1 can affect tRNA Cleavage in a Vacuole or Vacuole-like Compartment

An unresolved issue is how Rny1 is exposed to its RNA substrates during stress. One possibility, suggested by the loss of Rny1-GFP from vacuoles during stress [14], is that Rny1 is released to the cytoplasm and then can cleave various RNAs. Alternatively, or possibly in addition, RNAs might be transported into the vacuole by an autophagy-related process. One prediction of this latter model is that in the absence of Rny1, RNAs would be transported into vacuole or vacuole-like compartments, but they would not be degraded due to Rny1’s absence. Accordingly, increased accumulation of specific RNAs should be observed within biochemical fractions containing vacuoles.

**Figure 6:**
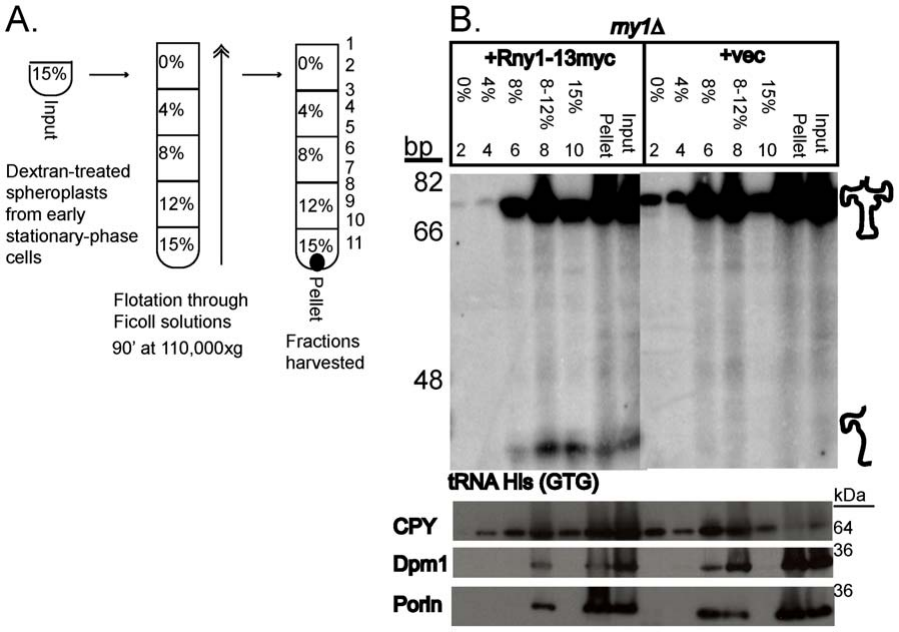
Rny1 cleaves tRNA at vacuoles. Equal amounts of cells were used to make dextran-treated spheroplasts of *rny1*Δ expressing Rny1 on a low-copy plasmid (Rny1–13myc) or vector (vec), prepared after one day of growth from midlog. These were floated on Ficoll gradients (0%, 4%, 8%, and 12% Ficoll solutions layered over samples in 15% Ficoll) to probe for Rny1’s impact on RNA cleavage at vacuoles, process is diagrammed in (A). Even-numbered fractions examined on Western blots and Northern blots. (B) *upper panel*. RNA from equal volumes of Ficoll fractions resolved by urea-acrylamide electrophoresis and Northern blotted for tRNA His (GTG). Input lanes were loaded at 50%. Position of fragments is indicated. Migration of oligonucleotide standards is shown in base pairs (bp). Numbers given above lanes represent fraction numbers on Ficoll gradients from top to bottom as diagrammed in (A) All samples are from the same image of the same blot, and the image was cropped after scaling the image to show only relevant samples. *lower panel*. Equal volumes of Ficoll fractions from the same experiment were denatured in buffer and resolved by SDS-PAGE. Input lanes were loaded at 50%. Western blots were performed for the indicated proteins to represent vacuoles (CPY, carboxypeptidase Y), ER (Dpm1), and mitochondria (Porin). Migration of molecular weight standards is indicated.

To test this possibility, we floated cell lysates from early stationary phase cells (where tRNAs are being cleaved by Rny1) on Ficoll step gradients (process diagrammed in Figure 6A). In this experiment, we compared *rny1*Δ strains either expressing Rny1 on a functional low-copy plasmid or an empty vector. RNA and proteins were prepared from equal volumes of each fraction and resolved by urea-acrylamide electrophoresis and SDS-PAGE, respectively. As expected for membrane compartments, we observed that ER (Dpm1) and mitochondrial markers (Porin) floated in the 8 and 8–12% fractions (although some remained in the input pellet). In contrast, we observed that the vacuolar marker Cpy1 was distributed across the gradient suggesting the presence of a diversity of vacuoles with different densities (Figure 6B). Consistent with this interpretation, we observed that intact vacuoles were present in each fraction as judged by staining with a vacuole specific dye (MDY-64) and examination of the fractions on a microscope. Given this, we conclude that vacuoles in yeast have a range of densities, distribute across the gradient, and the lightest fractions have the purest vacuole fractions due to the absence of other membrane-bound compartments [34].

Examination of tRNAs in the wild-type strain revealed that the tRNA cleavage products were distributed in the 8, 8–12, and 15% fractions (Figure 6B). The presence of these fragments in the 8 and 8–12% fractions suggests that they are either associated with, or within, a membrane compartment. The absence of these fragments from the lightest fractions suggests that if they are produced by Rny1 action within vacuoles, those vacuoles are of higher density. Interestingly, in the *rny1*Δ strain, we reproducibly observed an increased level of the full-length tRNA in the lightest fractions (Figure 6B). This suggests that the full-length tRNA can associate with light vacuoles, and is either fully degraded by Rny1 in that context, or such vacuoles mature to higher densities in wild -type strains. Thus, although we cannot rigorously determine the nature of the tRNA-membrane interactions, these observations demonstrate that both full-length and fragmented tRNAs can associate with membrane-bound compartments.

## Discussion

Our results reveal that multiple features of Rny1 contribute to its function in growth inhibition. Deleting either the signal sequence, the T2 conserved region, or the fungal C-terminal extension partially alleviated the growth defects with Rny1 over-expression (Figure 2A). These effects could not be attributed to a loss of protein expression (Figure 2B) since mutant proteins were expressed similarly to the wild-type protein.

In contrast, Rny1’s nuclease activity maps to specific loops within the conserved T2 region. Loss of the T2 region, but not the C-terminal extension, inhibited Rny1’s ability to cleave tRNA (Figure 3). Within this region, we identified specific loops required for cleavage of tRNA (Figure 5, loops 2, 3, 4, 6, and 7), two of which (loop 4 and loop 7) align to loops predicted to be involved in nucleotide binding (Figure 1).

Targeting to membrane compartments is important for Rny1’s cleavage of tRNA. Rny1’s signal peptide, presumably inserted into the ER during translation, is required for cleavage of tRNA (Figure 3). Several possibilities could be envisioned to explain the role of the signal peptide in regulation of Rny1’s functions. One possibility is that in the absence of ER targeting, loss of glycosylation disrupts interactions required for function. This is supported by our evidence that GFP-Rny1, which fails to be glycosylated (Figure 4B), also fails to cleave tRNA (Figure 4A) and partially rescues growth inhibition (data not shown). Another possibility is that the signal peptide directs Rny1’s vacuolar targeting, and this localization could enable processing of the Rny1 zymogen that renders it active. Lastly, it is possible that loss of the signal peptide could render an expressed but structurally impaired protein. In this case, our evidence further strengthens a model for a veritable catalytic-independent function for Rny1, requiring structural integrity, since a protein lacking the signal peptide partially alleviates growth inhibition (Figure 2A).

Our work raises the possibility that some tRNAs are taken up into vacuoles, or associate with vacuoles, for degradation by Rny1. Our analysis of vacuoles on Ficoll gradients revealed that specific fractions are enriched for CPY, a vacuolar protein, but not mitochondrial or ER proteins (Figure 6B, fractions 2 and 4, lower panels). These same vacuolar fractions clearly displayed tRNA in an *rny1*Δ strain (Figure 6B, fractions 2 and 4, upper panels), and expression of a functional form of Rny1 nearly eliminated detection of tRNA in these fractions (Figure 6B, fractions 2 and 4, upper panels).

The fragments of tRNA cleavage accumulated in denser fractions, containing CPY and other organellar markers. Our microscopic analysis of Ficoll fractions from these gradients revealed that intact vacuoles partition throughout the gradient (data not shown) consistent with the observation that CPY signal is found throughout the gradient. We speculate that tRNA fragments might associate with a denser form of vacuoles, perhaps associated with additional proteins recruited in an Rny1-dependent manner, which are not easily resolved from ER and mitochondrial markers. In the absence of Rny1, undigested tRNA might associate with lighter vacuoles more easily resolved from other cellular organelles.

Our evidence suggests that Rny1 might participate in a new form of tRNA ribophagy. To our knowledge, this is first example of tRNAs being targeted for vacuolar association or uptake. Prior work has demonstrated that rRNA accumulates within the lysosomes of neurons deficient for RNASET2 [15] and that ribosomal proteins traffic to vacuoles during autophagic conditions [35]. While tRNA cleavage does not require autophagy proteins [14], our work supports a model whereby tRNAs are turned over at, or within, vacuoles in a T2 ribonuclease-dependent manner during nutrient-limiting conditions, perhaps utilizing novel targeting mechanisms that do not include normal autophagy.

It is still possible that Rny1 can exit vacuoles to cleave RNA within the cytosol, in addition to acting as a nuclease within vacuoles. In previous work, our lab observed decreased vacuolar signal for the Rny1-GFP fusion protein during oxidative stress, supporting a model for translocation of Rny1 to the cytosol to contact RNA substrates [14]. This translocation resembles that observed for the predominant mitochondrial nuclease, Nuc1, which exits mitochondria during oxidative stress to modulate nuclear and possibly cytoplasmic RNA degradation [18]. Likewise, in mammalian cells, lysosomal cathepsins can enter the cytosol during oxidative stress and other stresses cumulating in cell death (reviewed in [19]). Hence, we speculate that in our studies of stationary phase conditions, Rny1 acts within vacuoles, and oxidative stress could trigger Rny1’s translocation similarly to Nuc1 and lysosomal cathepsins.

Our work provides two important implications for general T2 ribonuclease functions. First, our studies suggest that glycosylation and membrane targeting of T2 ribonucleases could be important for nuclease and toxic functions and could regulate self-recognition and immune cell interactions. In the case of RNASET2, whose catalytic mutants inhibit tumorigenesis by recruiting competent immune cells to tumor sites, plasma membrane trafficking (evidenced in [29]) and glycosylation of the T2 protein [36] might enable immune surveillance at the cell surface. Second, our studies suggest that tRNA, in addition to rRNA, might accumulate within acidic organelles of cells deficient for T2 ribonuclease function during stress. Since ribosomal proteins are also taken up into vacuoles and degraded during similar cellular stress [35], it is possible that translation complex proteins and RNA are turned over at vacuoles to control translation, through vacuolar protease and T2 ribonuclease functions. Thus, in diseases arising from RNASET2 deficiency, it is possible that regulation of protein synthesis is aberrant and drives pathogenesis.

## Materials and Methods

### Yeast Strains and Growth Conditions

Yeast strains and plasmids used in this study are described in Table 2. Cells were grown at 30°C in all experiments. For experiments over-expressing Rny1, pRP1584 and pRP1587 were used with pRP861 as a vector control. Cells were grown in selective synthetic media containing 2% sucrose to saturation, and these cultures were pelleted, aspirated, and diluted (OD600 = 0.1) in selective synthetic media with 2% galactose as the sole carbon source and grown to early midlog (OD600 = 0.3–0.5). For experiments to analyze tRNA fragment cleavage, cells were grown to saturation in media specified in figure legends (either selective synthetic media or yeast extract/peptone media both containing 2% dextrose), then diluted and grown to early midlog. Early midlog times were recorded, and cells were grown three days from this time to represent stationary phase growth. For frog ponding, yeast strains were patched to selective media plates, diluted to OD600 = 0.1 in selective media containing 2% sucrose in the first column of a 96-well plate, then diluted by 10-fold across into four additional columns. These columns were directly plated without additional growth.

**Table 2:** Yeast strains, plasmids, and oligonucleotides used in this study.

Strain/Plasmid/Oligo	Genotype/Description/Oligo sequence	Reference
BY4741	*Mat * ***a*** * his3D1 leu2D0 met15D0 ura3D0*	(Brachmann et al., 1998)
*hir2*Δ	*Mat * ***a*** * his3D1 leu2D0 met15D0 ura3D0; hir2*Δ*::kanMX4*	(Winzeler et al., 1999)
*rny1*Δ	*Mat * ***a*** * his3D1 leu2D0 met15D0 ura3D0; rny1*Δ*::kanMX4*	(Winzeler et al., 1999)
pRP1584	RNY1 under a *GAL* promoter on a 2µ URA3 plasmid, obtained fromThermo Scientific	(Thompson and Parker, 2009b)
pRP1587	pRP1584 mutated at two DNA sites encoding catalytic histidines tomake catalytically inactive protein	(Thompson and Parker, 2009b)
pRP861	Used as vector in experiments using GAL-RNY1. pPS293: GAL1 promoter(EcoRI/XbaI fragment from pPS231), 2µ,URA3	(Lee et al., 1996)
oRP1398tRNA His (GTG)	GTACTAACCACTATACTAAG	(Thompson and Parker, 2009b)
oRP1401tRNA Met (CAT)	GCGCCGCTCGGTTTCGATCC	(Thompson and Parker, 2009b)

### Plasmids and Plasmid Construction

Plasmids used in this study are listed in Table 2. The *GAL RNY1 2µ* plasmid (pRP1584) and its counterpart plasmid containing mutations to produce catalytically inactive Rny1 (pRP1587) were the templates utilized to generate *cis* mutants by PCR using primers for site-directed mutagenesis.

### RNA Analyses

Total RNA was prepared from liquid nitrogen flash frozen pellets. For all RNA analyses excepting that of Ficoll fractions, a hot acid phenol preparation was used. All steps prior to acid phenol addition were performed at 4°C. Samples were suspended in TNE buffer (50 mM Tris-Cl pH7.4, 100 mM NaCl, 10 mM EDTA), lysed with beads (two one-minute high speed vortexes interrupted by a one-minute incubation on ice to prevent overheating), then vortexed with SDS added to 1% and an equivalent volume of acid phenol chloroform. Vortexing was repeated, then samples were heated to 65°C for seven minutes, followed by additional vortexing. After acid phenol chloroform extraction, an additional acid phenol chloroform extraction, and one chloroform extraction, RNA was precipitated, washed, dried, and resuspended in deionized formamide. For Ficoll flotation assays, equal amounts of RNA were prepared (400 µl) using TriZol LS reagent (Invitrogen, Grand Island, NY, USA) following the manufacturer’s protocol, and pellets were resuspended in deionized formamide. Equal amounts of RNA (20 µg) as determined by A260, or equal amounts of RNA prepared from equal volumes (for analyses of floating RNA on Ficoll gradients), were resolved on 10% acrylamide, 47% urea, 1XTBE gels next to HinfI-digested, alkaline phosphatase treated, γ-^32^P-5′ end-labelled PhiX174 markers. Electrophoretic transfer to positively charged nylon membranes was performed in 0.5XTBE buffer. Blots were UV cross-linked twice, prewashed once at 65°C, using 0.1%SDS 0.1XSSC, then prehybridized in 6XSSC 0.1%SDS 10X Denhardt’s at 42°C. Hybridization with γ-^32^P-5′ end-labelled probes was performed in the prehybridization buffer. Blots were washed with 6XSSC 0.1%SDS and placed against phosphor screens to expose, and screens were scanned into a Typhoon scanner (GE Healthcare, Piscataway, NJ, USA) and quantitated using ImageQuant software.

### Protein Analyses

Lysates were prepared from harvested pellets of cells lysed using 5 M urea, boiled, then vortexed in glass beads for 5 minutes. A solution of 125 mM Tris-Cl pH6.8, 2% SDS was added at 2.5x the volume of 5 M urea used, and this was vortexed into the mixture, then samples were boiled a second time. Collected lysate was clarified by spinning at 16000 RCF, and the supernatant was harvested for analysis. Cleared lysates, or proteins analyzed from Ficoll fractions, were suspended in protein loading buffer (0.05 M Tris pH6.5, 1%SDS, 0.01% bromophenol blue, 10% glycerol), boiled, and run on a 10% Tris-SDS acrylamide gel next to SeeBlue Plus2 protein molecular weight standards (Invitrogen, Grand Island, NY, USA). Gels were transferred to nitrocellulose and probed using standard Western blotting protocols. Antibodies used were supplied by Invitrogen Molecular Probes, Grand Island, NY, USA (CPY, Dpm1, Porin), Novagen, Madison, WI, USA (His tag detection of Rny1), and Covance, Princeton, NJ, USA (GFP) all used with an anti-mouse secondary coupled to HRP (Sigma, St. Louis, MO, USA, #A4416). Signals were revealed using Pierce (Rockford, IL, USA) SuperSignal West Dura and exposing the blots to film and developing in a film processor (Konica, Mahwah, NJ, USA). Films were scanned into.tif format using an HP Scanjet Pro flatbed scanner (Hewlett Packard, Palo Alto, CA, USA), and images were analyzed using a cross-reactive band as a loading control.

### PNGase F Digests

Wild-type strains expressing Rny1-GFP or GFP-Rny1 were harvested at midlog after continuous growth in synthetic selective media containing galactose for expression. Total lysates were prepared and quantitated for proteins by Bio-Rad (Hercules, CA, USA) assay. Reactions utilized NEB reagents (Ipswich, MA, USA). Two reactions, each containing 20 µg of proteins, were prepared for each sample, diluting into glycoprotein denaturing buffer. Digests were first denatured (10′ at 100°C), then G7 reaction buffer, NP40, PNGase F or ddH20 were added according to the NEB protocol. Reactions were incubated at 37°C for 1 hour, then protein loading buffer was added, and samples were resolved by SDS-PAGE and analyzed by Western blot (performed as indicated in Protein analysis) probing for GFP (Covance Princeton, NJ, USA).

### Preparation of Vacuoles by Ficoll Flotation Gradients

Vacuoles were floated on Ficoll gradients using a modified version of the protocol described here [34]. For our experiments, we diluted 40 ml saturated starter cultures growing in synthetic media supplemented with complete amino acids and 2% dextrose (complete+dex) to 0.1 OD600/ml in 300 ml of fresh complete+dex. Cells were grown in 1L flasks shaking at 30°C, and the time of early midlog (0.3–0.4 OD600/ml) was noted. Twenty-four hours from this point, cells were harvested for analysis, and the OD600/ml was noted. Equal amounts of OD600 units (631) were harvested and processed for each sample. Cell walls were rendered susceptible to spheroplasting by resuspending in 30 ml of DTT solution (detailed in [34]) and incubating at 30°C for ten minutes, then harvested cells were spheroplasted using 18 ml of spheroplasting buffer containing 15,000 units of lyticase (Sigma) incubated at 30°C for 35′–45′. The A800 readings in water were observed and noted to determine optimal times for harvesting spheroplasts (10-fold reduction in A800), but all samples were harvested at the same time to avoid differences in processing. After harvesting spheroplasts, dextran removal of plasma membranes was performed as described [34] suspending in 15% Ficoll and adding 480 µl of 0.4 mg/ml DEAE Dextran (Sigma, St. Louis, MO, USA). In our experiments, we applied 3 ml of our lysate to the bottom of a pre-chilled SW40 tube (#331374 Beckman tubes), and we layered 2.5 ml of each of the following solutions in the following order on top: 12%, 8%, 4%, and 0%. Gradients were ultracentrifuged for 90 minutes at 110,000*xg* using a pre-chilled SW40 swinging bucket rotor. All steps following spheroplasting were performed in a 4°C room, and all tubes were prechilled. Following ultracentrifugation, 1 ml fractions were collected from the top and placed in protease inhibitors, using the 20xpic stock [34] diluted to 1x for RNA fractions. From each fraction, an equivalent volume was removed to another tube for protein analysis, and this tube was pre-loaded with chilled complete EDTA-free protease inhibitors (Roche, Basel, Switzerland) to yield 1X, prepared from a 7X stock in PS buffer. All of the samples, except for aliquots reserved on ice in a 4°C room for analysis of vacuoles and ribonuclease protection assays, were flash frozen in liquid nitrogen and stored at −80°C. Separate tubes for protein analysis and for RNA analysis ensured that thawing was only performed once (in a 4°C room) to prepare RNA or resolve proteins on gels. Purified vacuoles were examined using MDY-64 (Invitrogen Molecular Probes, Grand Island, NY, USA) label and found to be intact using the GFP filter on a Delta Vision microscope.
